# Exaggerated Exercise Blood Pressure as a Marker of Baroreflex Dysfunction in Normotensive Metabolic Syndrome Patients

**DOI:** 10.3389/fnins.2021.680195

**Published:** 2021-06-09

**Authors:** Akothirene C. Dutra-Marques, Sara Rodrigues, Felipe X. Cepeda, Edgar Toschi-Dias, Eduardo Rondon, Jefferson C. Carvalho, Maria Janieire N. N. Alves, Ana Maria F. W. Braga, Maria Urbana P. B. Rondon, Ivani C. Trombetta

**Affiliations:** ^1^Instituto do Coracao, Hospital das Clinicas HCFMUSP, Faculdade de Medicina, Universidade de São Paulo, São Paulo, Brazil; ^2^Universidade Metodista de São Paulo, São Paulo, Brazil; ^3^School of Physical Education and Sport, University of São Paulo, São Paulo, Brazil

**Keywords:** metabolic syndrome, cardiovascular risk, baroreflex sensitivity, muscle sympathetic nerve activity, cardiopulmonary exercise test, exercise blood pressure response

## Abstract

**Introduction:**

Exaggerated blood pressure response to exercise (EEBP = SBP ≥ 190 mmHg for women and ≥210 mmHg for men) during cardiopulmonary exercise test (CPET) is a predictor of cardiovascular risk. Sympathetic hyperactivation and decreased baroreflex sensitivity (BRS) seem to be involved in the progression of metabolic syndrome (MetS) to cardiovascular disease.

**Objective:**

To test the hypotheses: (1) MetS patients within normal clinical blood pressure (BP) may present EEBP response to maximal exercise and (2) increased muscle sympathetic nerve activity (MSNA) and reduced BRS are associated with this impairment.

**Methods:**

We selected MetS (ATP III) patients with normal BP (MetS_NT, *n* = 27, 59.3% males, 46.1 ± 7.2 years) and a control group without MetS (C, *n* = 19, 48.4 ± 7.4 years). We evaluated BRS for increases (BRS+) and decreases (BRS−) in spontaneous BP and HR fluctuations, MSNA (microneurography), BP from ambulatory blood pressure monitoring (ABPM), and auscultatory BP during CPET.

**Results:**

Normotensive MetS (MetS_NT) had higher body mass index and impairment in all MetS risk factors when compared to the C group. MetS_NT had higher peak systolic BP (SBP) (195 ± 17 vs. 177 ± 24 mmHg, *P* = 0.007) and diastolic BP (91 ± 11 vs. 79 ± 10 mmHg, *P* = 0.001) during CPET than C. Additionally, we found that MetS patients with normal BP had lower spontaneous BRS− (9.6 ± 3.3 vs. 12.2 ± 4.9 ms/mmHg, *P* = 0.044) and higher levels of MSNA (29 ± 6 vs. 18 ± 4 bursts/min, *P* < 0.001) compared to C. Interestingly, 10 out of 27 MetS_NT (37%) showed EEBP (MetS_NT+), whereas 2 out of 19 C (10.5%) presented (*P* = 0.044). The subgroup of MetS_NT with EEBP (MetS_NT+, *n* = 10) had similar MSNA (*P* = 0.437), but lower BRS+ (*P* = 0.039) and BRS− (*P* = 0.039) compared with the subgroup without EEBP (MetS_NT−, *n* = 17). Either office BP or BP from ABPM was similar between subgroups MetS_NT+ and MetS_NT−, regardless of EEBP response. In the MetS_NT+ subgroup, there was an association of peak SBP with BRS− (*R* = −0.70; *P* = 0.02), triglycerides with peak SBP during CPET (*R* = 0.66; *P* = 0.039), and of triglycerides with BRS− (*R* = 0.71; *P* = 0.022).

**Conclusion:**

Normotensive MetS patients already presented higher peak systolic and diastolic BP during maximal exercise, in addition to sympathetic hyperactivation and decreased baroreflex sensitivity. The EEBP in MetS_NT with apparent well-controlled BP may indicate a potential depressed neural baroreflex function, predisposing these patients to increased cardiovascular risk.

## Introduction

Metabolic syndrome (MetS) is the overlap of cardiovascular risk factors (i.e., dyslipidemia, hypertension, insulin resistance, and visceral obesity) (Grundy et al., 2005) and has been found to increase the incidence of cardiovascular events and death up to 78% (Gami et al., 2007). Among these factors, visceral obesity is the most prevalent (90–100%) (Drager et al., 2009; Cepeda et al., 2015). High amount of visceral adiposity is associated with endothelial dysfunction (Romero-Corral et al., 2010) and impaired autonomic control (Triggiani et al., 2019), mechanisms related to blood pressure (BP) control, and regulation of cardiovascular function during exercise (Tzemos et al., 2015).

In hypertensive patients, arterial baroreflex sensitivity (BRS) is reduced, and this impairment has been found to be associated with increased sympathetic drive and higher BP (Laterza et al., 2007; Wustmann et al., 2009). Regardless of the presence of hypertension, the pathophysiological substrates of visceral obesity, dyslipidemia, and insulin resistance may alter autonomic control, predisposing MetS patients to increased cardiovascular risk (Gami et al., 2007). In fact, MetS is characterized by sympathetic hyperactivation, even in patients with MetS who do not have hypertension (Grassi et al., 2005). Undoubtedly, sympathetic hyperactivation and decreased BRS are involved in the progression of the MetS to cardiovascular disease (Trombetta et al., 2010).

The BRS is an important sophisticated autonomic mechanism in the regulation of the cardiovascular system, designated to buffer beat-to-beat fluctuations in arterial BP. Arterial baroreceptors are mechanosensitive nerves, located in the adventitia of the carotid sinus and aortic arch. An increase in BP causes vascular distension, and the baroreceptor deformation exerts reflex bradycardia and sympathoinhibition, which results in a vasodilation reflex. On the other hand, the baroreflex deactivation causes tachycardia reflex and elicits sympathetic-mediated vasoconstriction reflex. The main role of baroreflex is to maintain BP at physiological levels (Chapleau et al., 2001; Laterza et al., 2007). In humans, arterial baroreflex has been evaluated using several laboratory techniques, mainly by quantifying the reflex responses induced by the injection of vasoactive drugs or selective external stimuli of the carotid baroreceptors. A more recent non-invasive method, based on the computational analysis of spontaneous fluctuations in systolic BP and RR intervals, consists of a sensitive, simple, and inexpensive procedure that allows the quantification of spontaneous BRS in real-life conditions (Parati et al., 2000; La Rovere et al., 2008).

Since exercise induces physiological stress, the analysis of HR and BP responses during maximal progressive exercise may be used as a simple tool to test the integrity of autonomic control to adjust cardiac output. In this context, in a previous study we observed that MetS patients with the comorbidity obstructive sleep apnea (OSA) had impaired HR recovery after maximal exercise, and this seemed to be partly due to the sympathetic hyperactivity in these patients (Cepeda et al., 2015).

There are a large number of studies suggesting that the response (EEBP) during cardiopulmonary exercise test (CPET) that occurs in individuals with normal resting BP is predictive of risk for new-onset hypertension (Miyai et al., 2000; Farah et al., 2009; Tzemos et al., 2015) and cardiovascular disease (Schultz et al., 2013; Keller et al., 2017). Endothelial dysfunction, decreased proximal aortic compliance, and increased exercise-related neurohormonal activation seem to be the main mechanisms underlying this prognosis (Tzemos et al., 2015). The intrinsic risk posed by EEBP may be still higher in MetS patients, as they have pathophysiological substrates that have been consistently associated with autonomic and vascular dysfunction (Trombetta et al., 2010; Shimabukuro et al., 2016; Rodrigues et al., 2017).

Albeit the clinical relevance of EEBP is related to an increased risk of onset hypertension (Miyai et al., 2000), the underlying pathophysiological mechanisms have yet to be fully elucidated. Therefore, this study aimed to assess both whether normotensive patients with MetS have EEBP during maximal CPET and whether autonomic dysfunction, represented by impaired BRS and increased muscle sympathetic nerve activity (MSNA), is involved in this response.

## Materials and Methods

### Study Population

In this prospective study, we recruited 72 newly diagnosed and unmedicated MetS patients from the Outpatient Unit of the Heart Institute (InCor), University of São Paulo Medical School, aged between 40 and 60 years, non-smokers, sedentary, with no history of alcohol consumption, and with no evidence of cardiopulmonary or skeletal muscle disorders. From the initial sample, the ones invited to participate in this study were only those 27 MetS (37.5%) without hypertension based on the office BP (SBP ≤ 139 and DBP ≤ 89 mmHg) (Mancia et al., 2013; Williams et al., 2019), and with normal BP confirmed by Ambulatory Blood Pressure Monitoring (ABPM: 24 h mean SBP/DBP < 130/80 mmHg; daytime < 135/85 mmHg; and nighttime < 120/70 mmHg) (Mancia et al., 2013; Williams et al., 2019), and we named this group as normotensive MetS (MetS_NT). Likewise, 19 age-matched subjects with normal BP (Williams et al., 2019), who presented only one or none of the other MetS risk factors, were enrolled in the study as a Control group (C). Part of these patients had also previously participated in other studies (Cepeda et al., 2015; Maki-Nunes et al., 2015; Rodrigues et al., 2017).

The study was approved by the Scientific Commission of the Instituto do Coracao (InCor), and by the Ethics in Research Commission of the Hospital das Clinicas HCFMUSP, Faculdade de Medicina, Universidade de São Paulo (#1222/05). All participants signed a written informed consent form.

### Procedures and Measures

#### Experimental Design

All evaluations were carried out in about 2–3 weeks on subsequent visits. The subjects were instructed to abstain from caffeine and physical activity for the 48 h leading up to the evaluations. Initially, venous blood was collected after 12 h of overnight fasting to measure total serum cholesterol, triglycerides, HDL cholesterol (enzymatic method), and plasma glucose (standard glucose oxidase method). After a light meal, all subjects underwent three standard BP measurements, and assessment of body weight, height, body mass index (BMI), and waist circumference (WC). Then, the 24-h ambulatory blood pressure monitoring (ABPM) was placed. The next day, ABPM was removed and the autonomic evaluation was performed in a quiet room with controlled temperature (22°C). In a lying position, the patient’s leg was positioned for microneurography, and a microelectrode was placed on the peroneal nerve. After instrumentation and a 15-min rest period, the biological signals of MSNA, heart rate (HR) on the electrocardiogram (ECG), and BP on a beat-to-beat basis were recorded for 10 min at rest in a lying position using a software program (WinDaq Software, Transonic Systems, DATAQ Instruments Inc., Akron, OH, United States). When we did not succeed in assessing MSNA, a second attempt was made on another date. On another visit, a CPET was performed.

Also, individuals underwent a night polysomnography to assess the apnea/hypopnea index (AHI).

#### Metabolic Syndrome Diagnosis

Metabolic syndrome patients were diagnosed according to Adult Treatment Panel III (ATP-III) (Grundy et al., 2005), which requires meeting at least three of the five following diagnostic criteria: (1) elevated WC ≥ 102 cm in men (≥40 inches) and ≥88 cm in women (≥35 inches); (2) elevated triglyceride ≥ 150 mg/dl (1.7 mmol/L); (3) reduced HDL-c < 40 mg/dl (1.03 mmol/L) in men and <50 mg/dl (1.3 mmol/L) in women; (4) elevated systolic BP (SBP) ≥ 130 mmHg and/or diastolic BP (DBP) ≥ 85 mmHg; and (5) elevated fasting glucose ≥ 100 mg/dl (5.6 mmol/L).

In the present study, we selected MetS patients without hypertension (Williams et al., 2019).

#### Office Blood Pressure

Systolic BP and DBP were measured following the recommended procedure for routine office BP measurement and with appropriate cuff size (Williams et al., 2019; Barroso et al., 2020).

#### Ambulatory Blood Pressure Monitoring

The 24-h BP measurements were recorded using a continuous blood pressure monitor (model 90207, Spacelabs Inc., Redmond, WA, United States). The device was placed on the non-dominant upper limb of the patient early in the morning and was removed 24 h later. The device was programed to record BP readings every 10 min during wake (daytime) and every 20 min during sleep (nighttime). To validate the ABPM, at least 70% of expected measurements should be conducted in 24 h recording (Williams et al., 2019). Daytime and nighttime intervals were defined using sleeping times reported by the patients in diary cards (awake and asleep periods) (O’Brien et al., 2013). The subjects were instructed to maintain their habitual activities and to relax and straighten out the arm during waking hour measurements. Thresholds for hypertension diagnosis based on ABPM were 24 h mean ≥ 130 and or ≥80 mmHg; awake (daytime) mean ≥ 135 and or ≥85 mmHg; and asleep (nighttime) mean ≥ 120 and or ≥70 mmHg (O’Brien et al., 2013; Williams et al., 2019).

#### Muscle Sympathetic Nerve Activity

Muscle Sympathetic Nerve Activity was directly measured through a multiunit postganglionic efferent from the peroneal nerve using the microneurography technique, which consists of the impaction of a tungsten microelectrode to the peroneal nerve, as previously described (Fagius and Wallin, 1993; Trombetta et al., 2010).

#### Beat-To-Beat Arterial Blood Pressure and Heart Rate

During 10 min at rest in a lying position, mean BP was continuously and non-invasively monitored by Finometer, a finger photoplethysmography device (Finapres 2300; Ohmeda, Englewood, Co., United States) on a beat-to-beat basis (WinDaq Software, Transonic Systems, DATAQ Instruments Inc., Akron, OH, United States) at a frequency of 500 Hz. Simultaneously, HR was continuously monitored through the lead II of the ECG. The respiratory rate was monitored with a piezoelectric thoracic belt (Pneumotrace II, model 1132) placed around the upper abdomen.

#### Spontaneous Baroreflex Sensitivity

A computer-based technique was used to evaluate the BRS by measuring spontaneous fluctuations beat by beat of BP and HR (see above) in the time domain. This method consists of the identification of three or more sequential beats, characterized by either a progressive rise in SBP and enlargement of the *R*–*R* interval (reflex bradycardia) or by a progressive decrease in SBP and shortening of the *R*–*R* interval (reflex tachycardia). The consecutive and simultaneous increases in SBP and increases in *R*–*R* intervals represent spontaneous activation of baroreceptors (BRS+), and consecutive and simultaneous decreases in SBP and decreases in *R*–*R* intervals represent spontaneous deactivation of baroreceptors (BRS−) (Parati et al., 2000; La Rovere et al., 2008). Basically, the values generated by the WinDaq Software were plotted on an excel spreadsheet, with the beat-to-beat SBP values (mmHg), and *R*–*R* intervals (from ECG) in milliseconds (ms). After plotting the values side by side, we observed each consecutive sequence of the SBP (from 1 mmHg) and *R*–*R* interval (from 3 ms), and a slope by a linear regression was generated. Finally, all slopes were averaged, resulting in an index of the sensitivity of arterial baroreflex modulation of heart rate (BRS). The cuff used was adapted according to the size of the patient’s finger and arm (Parati et al., 2000; La Rovere et al., 2008).

#### Blood Pressure Response to Exercise and Functional Capacity

Blood pressure response to exercise and functional capacity were measured using a maximal CPET on cycle ergometer fitted with an electromagnetic brake (Medifit 400L, Medical Fitness Equipment, Maarn, Netherlands) using a ramp protocol. The workload increments of 10 or 15 W were added every minute at constant cadence (60–70 rpm) until exhaustion. Oxygen uptake (VO_2_) and carbon dioxide production (CO_2_) were determined by gas exchange on a breath-by-breath basis in a computerized system (SensorMedics, model Vmax 229, BuenaVista, CA, United States). Peak VO_2_ was defined as the maximum VO_2_ reached at the end of the exercise (Beaver et al., 1986). We used the following criteria to define maximal effort achievement: (1) when the subject no longer maintained a cadence of 60 rpm and was unable to continue exercising, thus demonstrating exhaustion, and (2) when the maximal respiratory exchange ratio (RER) was greater than 1.10 (Balady et al., 2010). HR was continuously monitored during all tests by 12-lead electrocardiogram. Both SBP and DBP (auscultatory method) were measured at baseline, every 2 min of the exercise protocol, at peak, and at the first, second, and fourth minutes of recovery. The peak exercise SBP was considered the highest SBP value achieved during the CPET, and, consequently, the EEBP was defined as a peak exercise SBP ≥ 210 mmHg in men and ≥190 mmHg in women, according to the Framingham criteria (Tsioufis et al., 2008, 2012).

### Statistical Analysis

Statistical analysis was carried out using SPSS software (SPSS 20, Inc., Chicago, IL, United States). The data are presented as mean and standard deviation for parametric measurements and median and [interquartile range] for non-parametric measurements. The Kolmogorov–Smirnov and Levine tests were used to assess the normality and homogeneity of distribution of each variable studied. A Chi-square (χ^2^) test was used to assess categorical data differences. Comparisons between MetS_NT and C groups as well as between normotensive MetS with EEBP (MetS_NT+) and without EEBP (MetS_NT−) during CPET were carried out using Student’s *t*-test. The bivariate correlation (Pearson correlation) was conducted to test the association of peak SBP response during maximal CPET with baroreflex sensitivity (BRS−), association of triglycerides with peak SBP response during maximal CPET and association of triglycerides and BRS−. *P* < 0.05 values were considered statistically significant.

## Results

**FIGURE 1:**
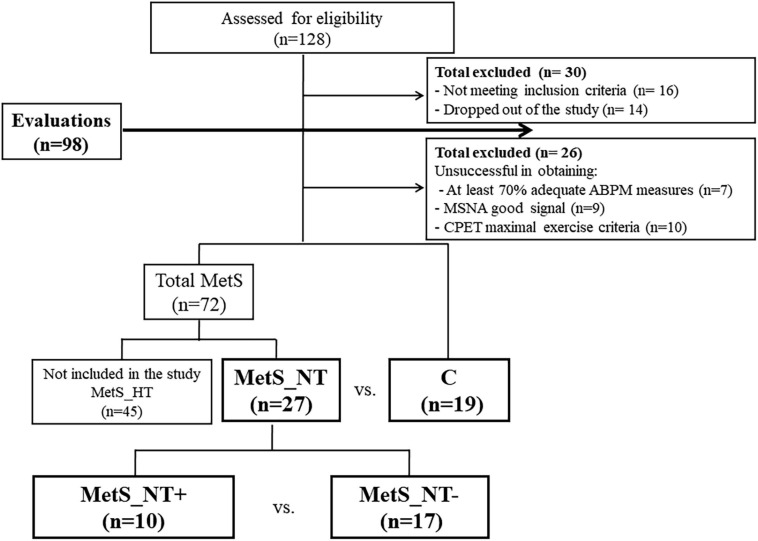
Participants flowchart. Note that there are two different parts in the study. First, we compared normotensive MetS patients (MetS_NT) with the control (C) group. Then, we compared the subgroup of MetS_NT that presented exaggerated exercise BP (MetS_NT+) with those without exaggerated exercise blood pressure response (MetS_NT−) during cardiopulmonary exercise test (CPET). ABPM, ambulatory blood pressure monitoring; MSNA, muscle sympathetic nerve activity.

Figure 1 depicts participants’ flowchart of the study, which has two different parts. First, we compared MetS_NT patients with the C group. Then, we compared the subgroup of MetS_NT that presented EEBP (MetS_NT+) with those without EEBP (MetS_NT−) during CPET.

### Normotensive MetS Patients vs. Control Group

**FIGURE 2:**
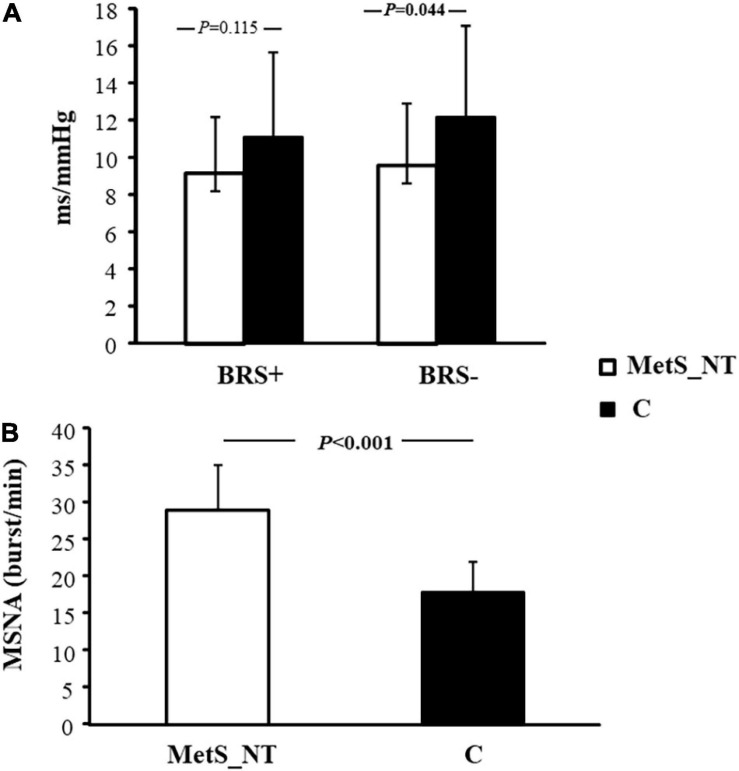
Baroreflex sensitivity for increases (BRS+) and decreases (BRS−) in spontaneous BP fluctuations **(A)**, and muscle sympathetic nerve activity [MSNA, **(B)**] in normotensive MetS patients (MetS_NT, *n* = 27) and control group (C, *n* = 19).

Table 1 displays physical characteristics, MetS risk factors, and AHI from polysomnography of the MetS_NT and C groups. Sex distribution, age, and functional capacity (peak VO_2_) were similar between groups. As expected, MetS_NT patients had higher weight and BMI and all MetS risk factors were impaired compared to C (Table 1). Regarding autonomic measurements, MetS_NT had lower BRS− (Figure 2A) and higher MSNA (Figure 2B) when compared to C. No difference was found in BRS+ (Figure 2A).

**TABLE 1:** Physical characteristics, metabolic syndrome (MetS) risk factors, and polysomnography measurements in normotensive metabolic syndrome patients (MetS_NT) and in control (C) group.

	**MetS_NT (*n* = 27)**	**C (*n* = 19)**	***P***
**Physical characteristics**			
Sex (M/F)	16/11	9/10	0.463
Age (years)	46 ± 7	48 ± 7	0.320
Weight (kg)	88.3 ± 12.6	70.2 ± 11.0	**<0.001**
BMI (kg/m^2^)	31.6 ± 3.9	25.3 ± 2.5	**<0.001**
Peak VO_2_ (ml/kg/min)	25.9 ± 6.4	25.6 ± 7.1	0.884
**MetS risk factors**			
WC (cm)	106 ± 8	90 ± 9	**<0.001**
Glucose (mg/dl)	102 ± 12	93 ± 8	**0.004**
Triglycerides (mg/dl)	199 [142–256]	98 [71–124]	**0.005**
HDL-c (mg/dl)	39 ± 9	51 ± 11	**<0.001**
Office SBP (mmHg)	117 [115–120]	111 [108–114]	**0.004**
Office DBP (mmHg)	80 ± 8	70 ± 9	**<0.001**
AHI (events/h)	14.5 [10–19]	8.0 [1–15]	0.091

*Data expressed as mean ± SD. BMI, body mass index; peak VO_2_, maximal oxygen uptake; WC, waist circumference; HDL-c, high-density lipoprotein cholesterol; SBP, systolic blood pressure; DBP, diastolic blood pressure; AHI, apnea/hypopnea index. Bold indicates the values that were significant (P < 0.05).*

Table 2 shows BP of the ABPM recordings and BP response during CPET in the MetS_NT and C groups. It should be noted that the 24-h, daytime, and nighttime means of SBP and DBP were similar in the MetS_NT and C groups, excepted for nighttime DBP, which was higher in MetS_NT. During CPET, MetS_NT had higher SBP and DBP at peak and at the first minute of recovery than C (Table 2).

**TABLE 2:** Ambulatory Blood Pressure Monitoring recordings (ABPM) and BP response during cardiopulmonary exercise test (CPET) in MetS_NT and in C group.

		**MetS_NT (*n* = 27)**	**C (*n* = 19)**	***P***
**Ambulatory blood pressure monitoring (ABPM)**				
SBP	24 h (mmHg)	117 ± 8	116 ± 9	0.670
	Daytime (mmHg)	123 ± 8	120 ± 9	0.366
	Nighttime (mmHg)	104 ± 8	107 ± 12	0.405
DBP	24 h (mmHg)	76 ± 7	78 ± 9	0.322
	Daytime (mmHg)	81 ± 8	82 ± 9	0.756
	Nighttime (mmHg)	71 ± 10	64 ± 7	**0.014**
**BP response during CPET**				
SBP	Peak (mmHg)	195 ± 17	177 ± 24	**0.007**
	1st min rec (mmHg)	188 ± 16	167 ± 21	**0.001**
DBP	Peak (mmHg)	91 ± 11	79 ± 10	**0.001**
	1st min rec (mmHg)	87[82–91]	78[73–83]	**0.007**

*Data expressed as Mean ± SD. SBP, systolic blood pressure; DBP, diastolic blood pressure. Bold indicates the values that were significant (P < 0.05).*

**FIGURE 3:**
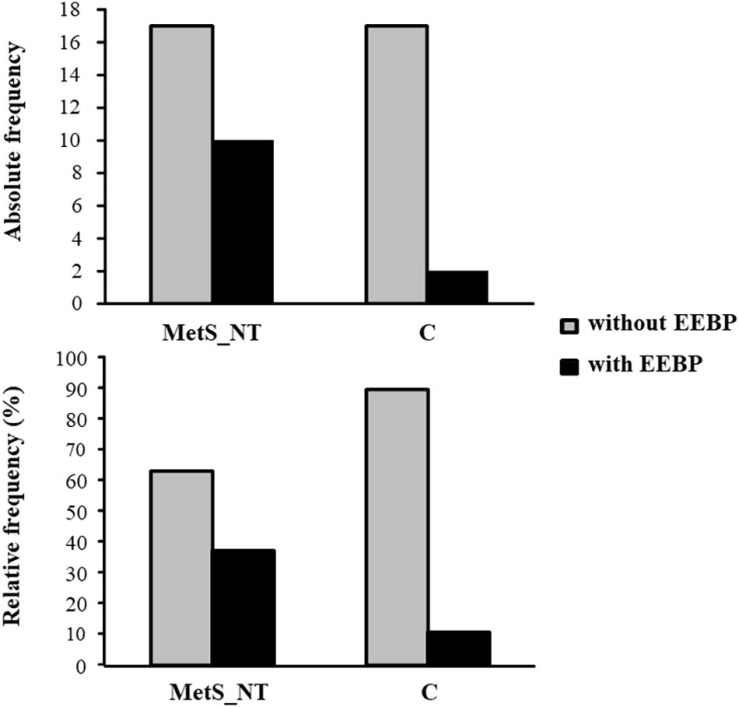
Absolute and relative frequency of exaggerated exercise blood pressure (EEBP = peak exercise SBP ≥ 210 mmHg in men and ≥190 mmHg in women) response during maximal CPET in the MetS_NT and in C. Note that the prevalence was significantly lower in the C group (*P* = 0.044). Ten (37%) of 27 MetS_NT presented EEBP during CPET, whereas only two in 19 individuals (10.5%) presented EEBP in the C group.

Figure 3 demonstrates the absolute and relative frequency of EEBP response during maximal CPET in MetS_NT and C. Ten (37%) of the patients with MetS_NT (four men and six women) presented EEBP during CPET (i.e., peak exercise SBP ≥ 210 mmHg in men and ≥190 mmHg in women), and we named this subgroup as MetS_NT+. On the other hand, the prevalence of EEBP in the C group was significantly lower (*P* = 0.044), with only two in 19 individuals (two men), i.e., 10.5% presented EEBP response (Figure 3).

### MetS With EEBP (MetS_NT+) Versus MetS Without EEBP (MetS_NT−)

In Table 3, we presented comparisons between MetS_NT+ (*n* = 10) and MetS_NT− (*n* = 17, without EEBP) regarding physical characteristics, office BP, BP of the ABPM recordings (24-h, daytime, and nighttime averages of SBP and DBP), and BP response during CPET. The subgroups MetS_NT+ and MetS_NT− were similar in sex distribution, age, weight, BMI, and peak VO_2_ (Table 3) and AHI (*P* = 0.127).

**TABLE 3:** Physical characteristics, office BP, BP of the ABPM recordings, and BP response during CPET in normotensive MetS with (MetS_NT+) and without (MetS_NT−) exaggerated exercise BP response (EEBP) during maximal CPET.

		**MetS_NT+ (*n* = 10)**	**MetS_NT**- **(*n* = 17)**	***P***
**Physical characteristics**				
Sex (F/M)		6/4	5/12	0.249
Age (years)		48 ± 6	45 ± 8	0.305
Weight (kg)		88.6 ± 10.4	88.1 ± 14.0	0.923
BMI (kg/m^2^)		33.1 ± 3.7	30.7 ± 3.8	0.114
Peak VO_2_ (ml/kg/min)		24.0 ± 6.8	27.0 ± 6.2	0.256
**Office BP measurements**				
Office SBP (mmHg)		120 ± 5	116 ± 7	0.089
Office DBP (mmHg)		81 ± 7	80 ± 6	0.620
**Ambulatory blood pressure monitoring (ABPM)**				
SBP	24 h (mmHg)	119 ± 7	115 ± 8	0.215
	Daytime (mmHg)	124 ± 7	122 ± 9	0.530
	Nighttime (mmHg)	107 ± 5	102 ± 9	0.153
DBP	24 h (mmHg)	76 ± 8	75 ± 7	0.906
	Daytime (mmHg)	81 ± 8	81 ± 8	0.811
	Nighttime (mmHg)	64 ± 7	64 ± 8	0.979
**BP response during CPET**				
SBP	Peak (mmHg)	210 ± 12	186 ± 14	**<0.001**
	1st-min rec (mmHg)	198 ± 14	182 ± 15	**0.025**
DBP	Peak (mmHg)	98 ± 11	87 ± 9	**0.007**
	1st-min rec (mmHg)	90[80–110]	85[70–105]	0.217

*Data expressed as mean ± SD. BMI, body mass index; peak VO_2_, maximal oxygen uptake; SBP, systolic blood pressure; DBP, diastolic blood pressure. Bold indicates the values that were significant (P < 0.05).*

Despite similar office BP (SBP and DBP), similar 24-h, daytime, and nighttime averages of SBP and DBP measured by the ABPM, these 10 MetS_NT+ who presented EEBP during CPET had higher SBP and DBP levels at peak of maximal exercise and higher SBP levels at the first minute of recovery (Table 3).

**FIGURE 4:**
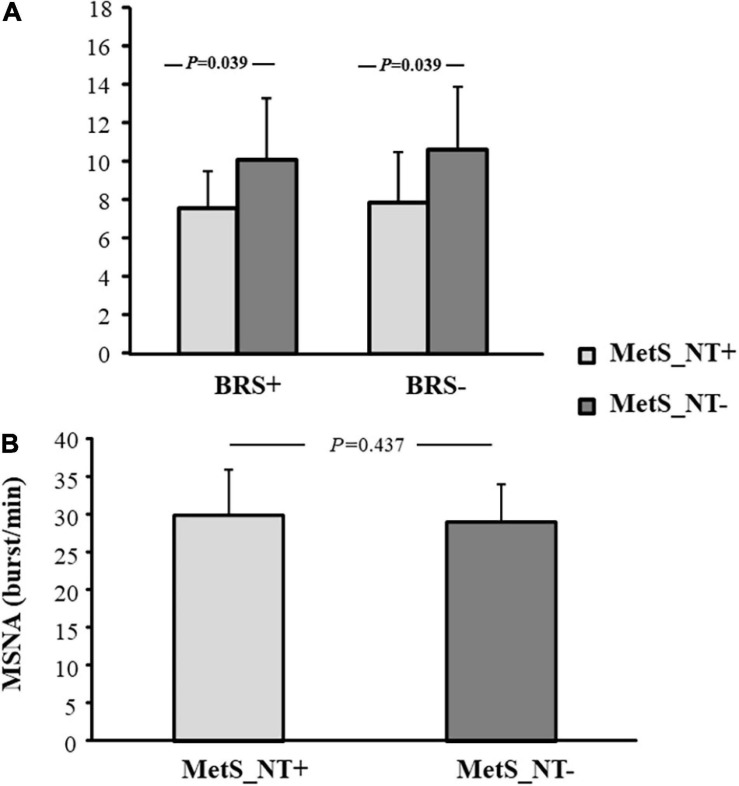
Baroreflex sensitivity for increases (BRS+) and decreases (BRS−) in spontaneous BP fluctuations **(A)**, and muscle sympathetic nerve activity [MSNA, **(B)**] in normotensive MetS patients who presented EEBP during maximal CPET (MetS_NT+, *n* = 10) and in normotensive MetS patients without EEBP during CPET (MetS_NT−, *n* = 17).

The MetS_NT+ subgroup had lower BRS+ and BRS− when compared to the MetS_NT− subgroup (Figure 4A), whereas MSNA was similar in the two subgroups (Figure 4B).

The univariate linear regression between peak SBP and BRS−, BRS+, and MSNA and between SBP at the first min of recovery and BRS−, BRS+, and MSNA in the MetS_NT subgroups with (MetS_NT+) and without (MetS_NT−) EEBP response during maximal CPET is shown in Table 4.

**TABLE 4:** Univariate linear regression between peak SBP and BRS−, BRS+, and MSNA and between SBP at 1st-min rec and BRS−, BRS+, and MSNA in normotensive MetS with (MetS_NT+) and without (MetS_NT−) EEBP response during maximal CPET.

		**Peak SBP**		**SBP 1st min rec**	
		***R***	***P***	***R***	***P***
	BRS+	−0.10	0.786	−0.41	0.312
MetS_NT+ (*n* = 10)	BRS−	−0.70	**0.024**	−0.73	**0.041**
	MSNA	0.40	0.283	0.56	0.148
MetS_NT− (*n* = 17)	BRS+	0.16	0.552	0.37	0.209
	BRS−	−0.02	0.933	0.28	0.361
	MSNA	0.19	0.558	−0.25	0.519

*BRS+, spontaneous baroreflex sensitivity for increases in BP; BRS−, spontaneous baroreflex sensitivity for decreases in BP; MSNA, muscle sympathetic nerve activity; SBP, systolic blood pressure; DBP, diastolic blood pressure. Bold indicates the values that were significant (P < 0.05).*

**FIGURE 5:**
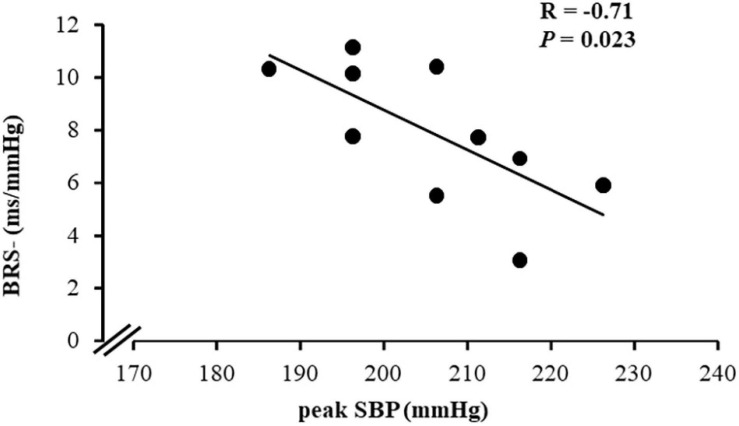
Correlation between peak systolic BP (peak SBP) during CPET and spontaneous baroreflex sensitivity for decreases in blood pressure (BRS−) in the subgroup of normotensive patients with MetS who presented exaggerated SBP during CPET (MetS_NT+, *n* = 10).

Interestingly, only in the MetS_NT+ subgroup, we found a strong negative correlation between peak SBP and BRS− (Table 4 and Figure 5). Similarly, in MetS_NT+ we found a negative correlation between SBP at the first minute at recovery and BRS− (Table 4).

In order to determine potential metabolic alterations related to impaired peak SBP during CPET, we analyzed the univariate and multivariate linear regression of all metabolic risk factors with peak SBP during CPET and with BRS− in the MetS_NT+ subgroup (Table 5). The univariate linear regression analysis showed a significant correlation between triglycerides with peak SBP and with BRS− (Table 5). For multivariate linear regression, we included all metabolic variables regardless of the previous “*P*” value in the univariate linear regression. In multivariate linear regression with peak SBP, no variable was found to be significant. On the other hand, with BRS− the triglycerides remained significant.

**TABLE 5:** Univariate and multivariate linear regression between peak SBP and metabolic risk factors and between BRS− and metabolic risk factors in the MetS_NT subgroups with EEBP (MetS_NT+) during maximal CPET.

**Peak**	**Univariate linear**		**Multivariate linear**		
**SBP**	**regression**		**regression**		
	***R***	***P***	**β**	**(95% CI)**	***P***
WC	0.10	0.794	0.06	(102–111)	0.877
Glucose	0.02	0.964	0.16	(94–107)	0.654
Triglycerides	0.66	**0.039**	0.68	(84–156)	0.195
HDL-c	−0.42	0.221	−0.04	(39–51)	0.934

**BRS−**	**Univariate linear**		**Multivariate linear**		
	**regression**		**regression**		
	***R***	***P***	**β**	**(95% CI)**	***P***

WC	0.45	0.188	0.218	(102–111)	0.367
Glucose	−0.36	0.301	−0.477	(94–107)	0.064
Triglycerides	0.71	**0.022**	−0.775	(84–156)	**0.034**
HDL-c	−0.31	0.382	−0.030	(39–55)	0.913

*BRS−, baroreflex sensitivity for decreases in spontaneous blood pressure; WC, waist circumference; HDL-c, high-density lipoprotein cholesterol. Bold indicates the values that were significant (P < 0.05).*

## Discussion

In the present study, we found that normotensive MetS patients exhibited higher values of peak systolic and diastolic BP during CPET when compared to normotensive controls, regardless of similar sex distribution, age, peak VO_2_, and AHI. In addition, they already presented sympathetic hyperactivation and decreased BRS−. Interestingly, some of these normotensive MetS patients (37%) had an EEBP response during maximal exercise (peak SBP ≥ 210 mmHg for men and ≥190 mmHg for women), in spite of similar office BP and similar averages of SBP and DBP at ABPM (24 h, daytime, and nighttime) when compared with normotensive MetS patients without EEBP response. Moreover, this prevalence of EEBP is higher in MetS_NT compared to C. We would like to point out that our MetS_NT subgroups, with or without EEBP response, were similar in age, sex, and functional capacity, factors that are known to impact EEBP (Schultz et al., 2015). However, the MetS_N subgroup that responds with EEBP (MetS_NT+) had lower BRS+ and lower BRS− than the subgroup without EEBP (MetS_NT−) (see Figure 4).

Our data corroborates a previous study conducted by Grassi et al. (2005). They found that sympathetic hyperactivation was not limited when MetS comprise hypertension as one of risk factors. However, unlike this previous study (Grassi et al., 2005), we found that patients with MetS with normal BP had attenuated BRS.

The major finding is that in the subgroup of normotensive MetS with EEBP response, the peak SBP was strong and negatively associated with BRS deactivation (see Figure 5). Therefore, the current study provides new and important findings on the autonomic mechanism involved in the EEBP response to exercise in these patients. Although office BP and BP in ABPM were similar (see Table 3), in this specific population with several metabolic risk factors the EEBP response denotes an arterial baroreflex dysfunction that seems to play a role in controlling BP during physiological stress, as it seems to do during exercise.

Notwithstanding using submaximal exercise and different measurement techniques as well as different SBP cutoff values to characterize EEBP, a recent community-based analysis from the Paris Prospective Study III (Sharman et al., 2018) has found similar results, observing that impaired BRS was independently associated with EEBP even among those with well-controlled resting BP. Nevertheless, this study that included 8,976 participants differs from ours in that the information pertaining to lifestyle and personal and family medical history (e.g., physical activity, disease status, and medication use) was achieved by self-administered questionnaires. Even so, this study reinforces that in normotensive subjects, decreased BRS seems to be a mechanistic pathway which may account for abnormal BP response to exercise.

It is well-established that adrenergic activation and baroreflex dysfunction are present in all hypertensive states (Grassi et al., 2007; Laterza et al., 2007; Seravalle et al., 2015). Likewise, exacerbation of BP during CPET is common in hypertensive patients (Schultz et al., 2015; Kader Abdel Wahab, 2016). Moreover, hypertensive patients with MetS had more than a twofold risk of exhibiting EEBP response than those without MetS (Tsioufis et al., 2012). Our aim in this study was to investigate whether this exacerbated BP response and autonomic changes were also detectable in the initial clinical phases of MetS, particularly in those patients without the presence of the hypertension component. Albeit it is consensus that decreased BRS is associated with increased sympathetic drive (Laterza, 2007) and higher BP (Wustmann et al., 2009), in our study only the BRS remained different between subgroups when we divided the normotensive MetS group (MetS_NT) by subgroups with (MetS_NT+) or without EEBP (MetS_NT−) response during maximal CPET, whereas MSNA did not. We cannot rule out that there was sympathetic hyperactivation at the peak of exercise in these patients with EEBP, since the measurement of MSNA was performed at rest, with the patients in a lying position. On the other hand, even in a small normotensive MetS group with EEBP response (*n* = 10), there was a strong negative association of peak SBP with BRS−, lending strength to the hypothesis that this autonomic reflex is an important pathophysiological mechanism underlying EEBP.

The mechanisms underlying an exacerbated BP response during exercise have yet to be fully understood and seem indeed to be multifactorial. So far, studies have pointed to endothelial dysfunction and changes in the intima-media thickness as the mechanisms underlying the exacerbation of BP during CPET in hypertension (Kader Abdel Wahab, 2016), perhaps caused by systemic vascular inflammation (Hamer and Steptoe, 2012). We may speculate that these alterations in turn may generate vascular resistance, causing arterial stiffness, and as such may be related to EEBP response in our sample. Indeed, in a previous study multivariate regression models clearly indicated that MetS, when compared to individual metabolic components, predicts impairment of endothelial dysfunction (Shimabukuro et al., 2016). To lend further support to this finding, a previous study conducted by our group involving MetS patients has demonstrated that impaired fasting glucose does play a role in vascular damage associated with sympathetic hyperactivation (Rodrigues et al., 2017). In another study with MetS patients, we found that the higher number of risk factors increase vascular dysfunction, as measured by pulse wave velocity (Lopes-Vicente et al., 2017). Additionally, besides age and increased SBP, altered triglycerides in the clustering worsened the stiffness of large vessels (Lopes-Vicente et al., 2017).

When hypertension is eliminated, controversy remains regarding the pathophysiological change and the EEBP response. In addition, only a few studies have focused on the effects of MetS on BRS (Grassi et al., 2005; Trombetta et al., 2010; Assoumou et al., 2012; Sharman et al., 2018). Sharman et al. (2018) demonstrated that impaired BRS, but not carotid stiffness, was independently associated with exaggerated exercise BP among those with well-controlled resting BP.

Our MetS population sample comprised recently diagnosed and unmedicated MetS patients without hypertension, a very common condition in middle-aged individuals who often do not consider themselves to be diseased. Our study brings a new perspective in that it lends prominence to the role of BRS as a key mechanism which may underlie the response of BP to CPET.

While elucidating the risk factors that impair autonomic control of BP during exercise in normotensive patients with MetS is indeed complex, given that these factors share pathophysiological pathways and seem to potentiate each other, the association of triglycerides with peak SBP may suggest the potential of this risk factor over some pathophysiological changes involved in hemodynamic control. However, the overlap of metabolic risk factors seems to be the main cause of the autonomic alteration in normotensive MetS patients.

In general, the overlap of several risk factors seems to be stronger in predicting the pathophysiological alterations related to attenuation of BRS as well as to EEBP response. Assoumou et al. (2012) have shown that decreased BRS slope was significantly correlated with MetS. Nevertheless, this association also independently was found for triglycerides and waist circumference in elderly patients with MetS (Assoumou et al., 2012).

Indeed, the overlap of several metabolic risk factors is the probable trigger to higher values of peak systolic BP during exercise, as shown by a previous study conducted by Tsioufis et al. (2012). They found that MetS in newly diagnosed hypertensive patients was associated with increased peak exercise BP and a higher frequency of EEBP, regardless of ambulatory BP levels and anthropometric characteristics. Our results extend this knowledge to the change in BRS in MetS without hypertension, which leads to the EEBP response that occurs beyond hypertension, predisposing these normotensive MetS with EEBP response to higher cardiovascular risk.

We have previously observed that OSA, a common comorbidity in patients with MetS, leads to a further impairment in autonomic control (Trombetta et al., 2010, 2013; Toschi-Dias et al., 2013; Cepeda et al., 2015). However, in the present investigation patients with hypertension as one of MetS risk factors were excluded, and our studied groups were similar in the AHI. Thus, OSA apparently did not affect BP response during exercise in these normotensive MetS patients, not even those with EEBP response.

Although several methods have been developed to study baroreflex function in humans, most of these techniques are of limited value for a daily practice in the clinical setting (La Rovere et al., 2008). Given that the BP measurement is a standard evaluation procedure during the graded exercise test, a routinely in-clinic exam, we may suggest the use of the Framingham criteria cutoff for peak SBP (≥210 mmHg in men and ≥190 mmHg in women) (Tsioufis et al., 2008, 2012) as a surrogate method to indicate probable baroreflex dysfunction in normotensive MetS patients.

In conclusion, the present study offers a new insight on the mechanisms underlying EEBP response to exercise and more particularly on the key role of BRS impairment in the increased BP response during exercise in normotensive patients with MetS. Our findings emphasize the importance of identifying EEBP response to exercise in MetS patients, even in those within the normal range of clinical BP, since exaggerated BP during CPET was associated with the baroreflex in MetS patients, which put these patients at a greater cardiovascular risk. Thus, an awareness of EEBP during maximal CPET in normotensive MetS patients should be part of any clinical interventions.

### Limitations

There are some limitations in our study that merit discussion. Previous studies addressing the mechanisms underlying EEBP have been carried out with healthy volunteers. The association of risk factors (at least 3) in our sample of normotensive patients with MetS can cause interpretation bias, since any of the factors, according to their prevalence, may be involved in the EEBP during CPET (Thanassoulis et al., 2012).

We would like to point out that it has already been demonstrated that women have distinct pathophysiological pathways from men (Lambert et al., 2007; Lew et al., 2017), as well as increased adiposity along with differences in endothelial function and recruitment of inflammatory cells. Even though our sample is composed of male and female patients, this should not affect our results, since we did not find any differences in gender distribution between the studied groups.

In this study, the office BP was measured in a single visit. According to the “Brazilian Guidelines of Hypertension–2020” (Barroso et al., 2020), true hypertension is defined when systematically abnormal BP values are measured in the office, or more assertively by using out-of-office measurements by ABPM. Following the guidelines, in order to confirm hypertension, our patients underwent an ABPM.

Regarding the method used for determining the BRS, the venous vasoactive drug infusion technique has been largely used. However, the lack of selectivity in the response has been considered as one important limitation of the use of vasoactive drugs (La Rovere et al., 2008). Thus, we opted for the spontaneous method, since it reflects spontaneous beat-to-beat baroreflex at rest while providing a safe and reliable non-invasive assessment of human BRS. However, the sequence method used in the present study is just one of the spontaneous BRS methods in literature and other methods may quantify different aspects of the autonomic BRS control (Silva et al., 2019). Another limitation is that a spontaneous method used to study the arterial baroreflex estimates the feedback effects of SBP changes on pulse interval (PI) and reciprocal of heart rate, neglecting the simultaneously occurring feedforward effects of PI on SBP, induced through changes in cardiac output (Parati et al., 2019).

Assessing auscultatory BP during treadmill maximal exercise is sometimes difficult. We took great care to have reliable peak BP measurement, using a stationary electromagnetic brake cycle ergometer.

## Data Availability Statement

The original contributions presented in the study are included in the article/Supplementary Material, further inquiries can be directed to the corresponding author.

## Ethics Statement

The studies involving human participants were reviewed and approved by Scientific Commission of the Instituto do Coracao (InCor), and by the Ethics in Research Commission of the Hospital das Clinicas HCFMUSP, Faculdade de Medicina, Universidade de São Paulo (#1222/05). The patients/participants provided their written informed consent to participate in this study.

## Author Contributions

ACD-M, SR, FXC, and ICT: conception and design of the work, data acquisition, analysis, interpretation of data, drafting of the work, and critical revision for important intellectual content. ET-D, ER, JCC, MJNNA, AMFWB, and MUPBR: data acquisition, interpretation of data for the work, and critical revision for important intellectual content. All authors read and approved the final version to be published, and all authors agreed with all aspects of the work in order to ensure the accuracy and integrity of the work.

## Conflict of Interest

The authors declare that the research was conducted in the absence of any commercial or financial relationships that could be construed as a potential conflict of interest.

## Supplementary Material

Supplementary Table 1 Univariate linear regression between peak SBP and metabolic syndrome (MetS) risk factors in all studied groups.

## Abbreviations

MetS: metabolic syndrome
C: control group without MetS
MetS_NT: normotensive MetS patients
CPET: cardiopulmonary exercise test
EEBP: exaggerated exercise blood pressure response during maximal cardiopulmonary exercise test, i.e., SBP ≥ 190 mmHg for women and ≥ 210 mmHg for men
MetS_NT+: normotensive MetS subgroup with EEBP response during CPET
MetS_NT−: normotensive MetS subgroup without EEBP response during CPET
BP: blood pressure
SBP: systolic BP
DBP: diastolic BP
HR: heart rate
BRS: spontaneous baroreflex sensitivity
BRS+: BRS for increases in spontaneous BP and HR fluctuations
BRS−: BRS for decreases in spontaneous BP and HR fluctuations
MSNA: muscle sympathetic nerve activity
ABPM: ambulatory blood pressure monitoring
OSA: obstructive sleep apnea
AHI: apnea/hypopnea index
BMI: body mass index
WC: waist circumference
ECG: electrocardiogram
VO_2_: oxygen uptake.
