# Supplementary material for: Exaggerated Exercise Blood Pressure as a Marker of Baroreflex Dysfunction in Normotensive Metabolic Syndrome Patients
Source: Front Neurosci. 2021 Jun 9;15:680195. doi: 10.3389/fnins.2021.680195 (PMC8219920; doi:10.3389/fnins.2021.680195)
Supplement: Supplementary Table 1 — Univariate linear regression between peak SBP and metabolic syndrome (MetS) risk factors in all studied groups. [file Data_Sheet_1.PDF]

Table. Univariate linear regression between peak SBP and metabolic syndrome (MetS) risk factors in total normotensive MetS group (MetS_NT), control group (C) and in subgroups of MetS_NT with (MetS_NT+) and without (MetS_NT-) exaggerated blood pressure response during maximal cardiopulmonary exercise test (CPET).

		**Peak SBP**	
		**R**	***P***
**MetS_NT (n=27)**	**WC**	0.27	0.171
	**Glucose**	-0.19	0.341
	**Triglycerides**	-0.13	0.517
	**HDL-c**	0.07	0.745
	**SBP**	0.36	0.067
	**DBP**	-0.51	**0.007**
**C (n=19)**	**WC**	0.47	0.065
	**Glucose**	0.19	0.427
	**Triglycerides**	-0.11	0.642
	**HDL-c**	-0.17	0.480
	**SBP**	0.35	0.152
	**DBP**	0.21	0.392
**MetS_NT+ (n=10)**	**WC**	-0.10	0.794
	**Glucose**	0.02	0.964
	**Triglycerides**	0.66	**0.039**
	**HDL-c**	-0.43	0.221
	**SBP**	0.52	0.125
	**DBP**	0.34	0.338
**MetS_NT- (n=17)**	**WC**	0.46	0.065
	**Glucose**	-0.20	0.439
	**Triglycerides**	0.19	0.464
	**HDL-c**	-0.52	**0.034**
	**SBP**	0.08	0.763
	**DBP**	0.36	0.161

WC, waist circumference; SBP, systolic blood pressure; DBP, diastolic blood pressure.
